# Exploratory study of selected nucleotide variants in *GRIN1*, *GRIN2A* and *GRIN2B* encoding subunits of the NMDA receptor in a targeted group of schizophrenia patients with chronic cognitive impairment

**DOI:** 10.1007/s43440-020-00192-1

**Published:** 2020-11-25

**Authors:** Marek Krzystanek, Marek Asman, Joanna Witecka, Artur Pałasz, Ryszard Wiaderkiewicz

**Affiliations:** 1 Department and Clinic of Psychiatric Rehabilitation, Faculty of Medical Sciences, Medical University of Silesia in Katowice, Ziołowa 45/47, 40-635 Katowice, Poland; 2 Department of Psychiatry and Psychotherapy, Faculty of Medical Sciences, Medical University of Silesia in Katowice, Ziołowa 45/47, 40-635 Katowice, Poland; 3 Department of Parasitology, Faculty of Pharmaceutical Sciences in Sosnowiec, Medical University of Silesia in Katowice, Jedności 8, 41-200 Sosnowiec, Poland; 4 Department of Histology, Faculty of Medical Sciences, Medical University of Silesia in Katowice, Medyków 18, 40-752 Katowice, Poland

**Keywords:** Schizophrenia, NMDA receptor, Cognitive deficits, Single nucleotide variants

## Abstract

**Background:**

Schizophrenia is a mental disease that affects approximately 1% of the population. Despite over 100 years of research, its pathomechanism has still not been clarified. Cognitive deficits, which are one of the symptomatic dimensions of schizophrenia, usually appear a few years before the first psychotic episode. Therefore, this is why they are probably the clinical manifestation of the primary pathomechanism of schizophrenia. It is also supposed that *N*-methyl-d-aspartate receptor (NMDA-R) insufficiency in the prefrontal cortex is responsible for cognitive deficits in schizophrenia. The study aimed to examine whether four selected single nucleotide variants in *GRIN1*, *GRIN2A* and *GRIN2B* encoding NMDA-R subunits, of which two have not been tested before, are linked with the selected clinical phenotype of cognitive dysfunction in schizophrenia.

**Methods:**

The study included the targeted group of 117 patients diagnosed with schizophrenia, all with cognitive deficits and in symptomatic remission. DNA fragments including the studied polymorphisms of the NMDA receptors subunit genes were amplified by polymerase chain reaction and subjected to sequencing.

**Results:**

The study did not confirm the presence of any of the four selected single nucleotide variants in *GRIN1*, *GRIN2A* and *GRIN2B* subunits of NMDA-R.

**Conclusions:**

The finding indicates that selected single nucleotide variants in GRIN2A and GRIN2B encoding subunits of the NMDA receptor are not associated with the presence of cognitive deficits in schizophrenia.

## Introduction

The pathomechanism of schizophrenia remains an unexplained mystery of psychiatry. Probably either the etiology of schizophrenia is complex, or it is a group of diseases with different pathomechanisms, clinical picture and prognosis. High expectations while solving the puzzle of schizophrenia were linked with results of resequencing the human genome. Unfortunately, the results of genetic testing so far are disappointing. It is estimated that the cumulative effect of single nucleotide variants (SNVs) explains only about 30% of the genetic risk of developing schizophrenia [1]. Nowadays, more attention is paid to the greater role played by mechanisms mediating the interaction between environmental factors and the regulation of gene expression in the pathogenesis of schizophrenia.

Since cognitive deficits in schizophrenia manifest several years before the period of acute psychosis, they are supposed to be symptoms of the primary pathogenic process of schizophrenia [2, 3]. In most patients, cognitive deficits begin 3–4 years before the first episode of the disease, i.e., in the prodromal period and do not occur in earlier periods of life [4]. Furthermore, long-term observations of schizophrenia indicate chronic persistence of cognitive deficits in patients [5, 6].

**Fig. 1:**
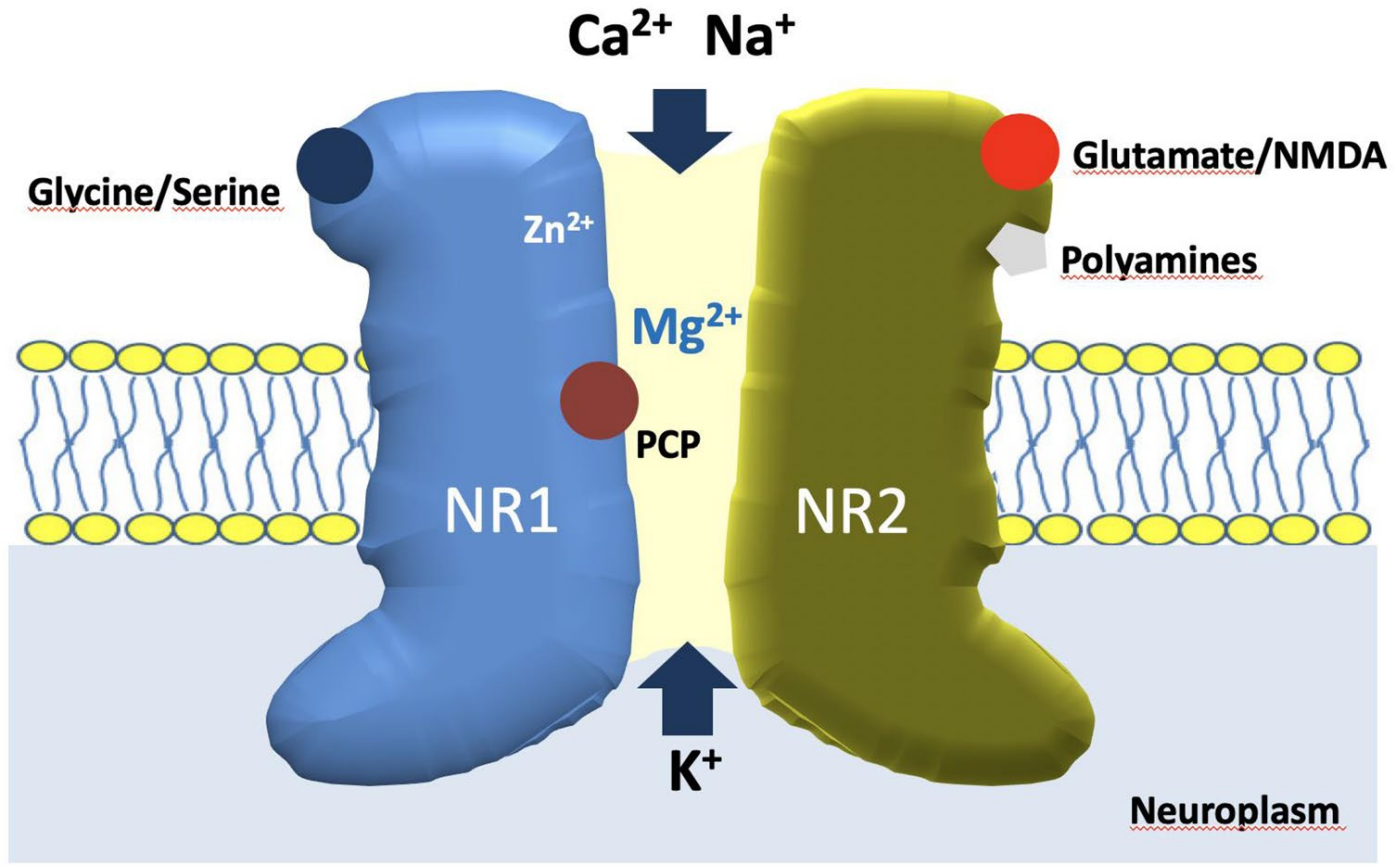
Schematic representation of NMDA receptor molecule anchored in the neural cell membrane. The main binding sites for important ligands (glutamate, glycine) and modulators such as zinc ions, polyamines and phencyclidine (PCP) are present in the structure of NR1 and NR2 subunits. The unique magnesium ion-dependent blockage of voltage-gated cationic channel plays a crucial role in the NMDAR function

Schizophrenia results from disturbances in the local and cortical-subcortical neuronal systems, involving a greater number of brain structure, and one of the components of the complex pathogenesis of this disease is a dysfunction of *N*-methyl-d-aspartate receptors (NMDA-Rs, Fig. 1) [7]. Involvement of NMDA-Rs in the pathogenesis of schizophrenia is indicated by the effect of blocking these receptors in healthy volunteers. Administration of NMDA-Rs’ antagonist ketamine causes psychotic, negative and cognitive symptoms characteristic of schizophrenia. Similarly, administration of ketamine to schizophrenic patients increases their psychotic, cognitive and negative symptoms [2]. This may indicate that inhibition of NMDA-R activity in the brain is of key importance for the development of cognitive deficits in schizophrenia, while dysregulation of the dopaminergic or serotonergic system is of secondary nature.

The hypothesis of NMDA-R’s deficiency as the main pathogenetic component responsible for schizophrenia was proposed by Carlsson [8, 9]. This model is related to the neurodevelopmental hypothesis of schizophrenia, which assumes that glutaminergic disturbances in various areas of the brain, including the prefrontal cortex, are responsible for the psychotic, cognitive and emotional symptoms [10]. According to the neurodevelopmental hypothesis of schizophrenia, disruption of glutaminergic system function in the developmental period is responsible for the prepsychotic symptoms of schizophrenia [3]. Then, in the period of late adolescence, glutaminergic hyperactivity occurs and this condition leads to full-symptomatic schizophrenia [3].

The insufficiency of NMDA receptors is closely associated with the appearance of cognitive dysfunctions and hypofrontality in schizophrenia [11]. Cognitive disturbances in schizophrenia, mainly in the form of attention and working memory deficit, result from prefrontal cortex dysfunction and are associated with glutaminergic transmission deficiency caused by NMDA-Rs hypofunction [12]. There is a lot of experimental and clinical evidence confirming the glutaminergic hypothesis of schizophrenia and the important role NMDA-Rs play in it [13]. In addition, NMDA-R function may be also responsible for hypofrontality induced by antipsychotics alone [14].

Numerous sequence variations, despite previously reported mutations, were identified in *GRIN1* gene. But their associations with selected clinical phenotype of cognitive deficit in schizophrenia and other psychiatric syndromes need to be still confirmed. Rice et al. [15] identified several variants in the *GRIN1* gene, including the promoter region (rs1114620 [1001G-C change]), in patients with schizophrenia (181,500). Begni et al. investigated the potential role of the 1001G-C variant in susceptibility to schizophrenia (181,500) in a study of 139 Italian patients with schizophrenia and 145 healthy control subjects [16]. Sequence analysis revealed that the C allele may alter the consensus sequence for the transcription factor NF-kappa-B (164,011). The frequency of this allele was higher in patients than in control subjects (*p* = 0.0085). The genotype distribution of the C allele was also different, with *p* = 0.034; if the C allele was considered dominant, the difference was more significant, *p* = 0.0137. Begni et al. concluded that *GRIN1* is a good candidate gene for susceptibility to schizophrenia [16].

Zhao et al. genotyped 5 SNVs in *GRIN1* in 2455 schizophrenic and non-schizophrenic Han Chinese subjects, including population- and family-based samples, and performed case–control and transmission disequilibrium test (TDT) analyses [17]. A highly significant association with schizophrenia was detected at the 5-prime end of *GRIN1*. Analysis of single variants and multiple-locus haplotypes indicated that the association is mainly generated by rs11146020 (case–control study: *p* = 0.0000013, OR = 0.61, 95% CI 0.50–0.74; TDT: *p* = 0.0019, *T*/NT = 79/123). Therefore, in this work, we decided to look for another not studied yet variant in the sequence of *GRIN1*, rs11146020/NG_011507.1:g.4476G > C/5′UTR in association with the selected clinical phenotype of cognitive deficit in schizophrenia.

In contrast to GRIN1, no SNVs in *GRIN2A* gene have been reported, in OMIM, in associations with schizophrenia and particularly with a clinical phenotype of cognitive deficit in schizophrenia. Swanger et al. assessed variations across *GRIN2A* (138253) and *GRIN2B* domains and determined that the agonist-binding domain, transmembrane domain, and the linker regions between these domains were particularly intolerant to functional variation [18]. Notably, the agonist-binding domain of *GRIN2B* exhibited significantly more variation intolerance than that of *GRIN2A*. To understand the ramifications of missense variation in the agonist-binding domain, the authors investigated the mechanisms by which 25 rare variants in the *GRIN2A* and *GRIN2B* agonist binding domains dysregulated NMDA receptor activity. When introduced into recombinant human NMDA receptors, these rare variants identified in individuals with neurologic disease had complex, and sometimes opposing, consequences on agonist binding, channel gating, receptor biogenesis, and forward trafficking. The approach combined quantitative assessments of these effects to estimate the overall impact on synaptic and non-synaptic NMDAR function. Interestingly, similar neurologic diseases were associated with both gain- and loss-of-function variants in the same gene. Most rare variants in *GRIN2A* were associated with epilepsy, whereas *GRIN2B* variants were associated with intellectual disability with or without seizures.

In summary, none of the variants reported to the SNP data bases were associated with the selected clinical phenotype of cognitive deficit in schizophrenia. An analysis of the four SNVs in the three genes encoding the NMDA-Rs subunits prompted us to aim on verifying whether persistence of cognitive dysfunctions despite the remission of other symptoms of schizophrenia may be a schizophrenia clinical phenotype associated with selected single nucleotide variants in the genes of NMDA-Rs’ subunits.

## Materials and methods

### Study group

A targeted group of patients diagnosed with schizophrenia (according to ICD-10 criteria and DSM-IV) was selected for the study. All patients were Caucasian. The group constituted a selected clinical phenotype of patients successfully treated for schizophrenia who, despite treatment, still have cognitive impairment. Patients were recruited in mental health outpatient’s clinics and psychiatric wards in the years 2007–2011. The study included patients who met the criteria of the clinical phenotype established in the study: the presence of cognitive impairment despite symptomatic remission of schizophrenia. All patients enrolled in the study met the following symptomatic remission criteria, confirmed by psychiatric examination and PANSS assessment [19]:

- severity of symptoms on the Positive and Negative Syndrome Scale (PANSS) scale ≤ 3 on the following scale symptoms: delusions (P1), unusual thought content (G9), hallucinatory behavior (P3), conceptual disorganization (P2), mannerisms and posturing (G5), blunted affect (N1), passive/apathetic social withdrawal (N4), lack of spontaneity and conversation flow (N6); (letter and numeric codes given next to the symptoms correspond to the numbers of symptoms on the PANSS scale),
- stable maintenance of symptoms at a level that allows daily functioning (severity in PANSS ≤ 3) for at least 6 months.

Of 247 patients included in the screening, 117 were enrolled. Moreover, 30 healthy volunteers aged over 45 were included in the study. Patients diagnosed with schizophrenia were treated as monotherapy with one of the following antipsychotics: haloperidol, olanzapine or clozapine.

The characteristics of the study groups are summarized in Table 1.

**Table 1:** Demographic and clinical description of the study groups

Patients	Schizophrenia patients			Healthy volunteers (*n* = 30)
	Antipsychotic drug (*n*—number of patients)			
	Haloperidol (*n* = 32)	Clozapine (*n* = 40)	Olanzapine (n = 45)	
Gender				
F—females	F = 15	F = 15	F = 21	F = 16
M—males	M = 17	M = 25	M = 24	M = 14
Age^a^ (± SD^b^)	52.3 (± 9.6)	43.5 (± 7.1)	41 (± 12)	57 (± 7.3)
Min–max^c^	37–65	34–53	22–61	46–65
Age of onset^a^ (± SD)	31 (± 8.5)	25.9 (± 8.2)	26.9 (± 6.1)	NA^d^
Min–max	22–38	18–41	20–39	
Duration of disease^a^ (± SD)	20.6 (± 11.9)	19.6 (± 6.4)	16 (± 7.9)	NA^d^
Min–max	6–40	6–29	1–31	
Number of hospitalizations	15.6	6.3	4.5	NA^d^
Dose mg/day	4.7 p.o.^e^ 67.3 LAI^f^ i.m.^g^ every 2–4 weeks	327.2	13.2	NA^d^

Patients in the study were taking one of the antipsychotic drugs: haloperidol, clozapine, or olanzapine. Haloperidol was taken in the form of tablets or long acting injection. The demographic characteristics of the healthy volunteers are also provided

^a^Years, ^b^standard deviation, ^c^minimum and maximum, ^d^not applicable, ^e^oral, ^f^long acting injection, ^g^intramuscular injection

### Cognitive dysfunctions

**Fig. 2:**
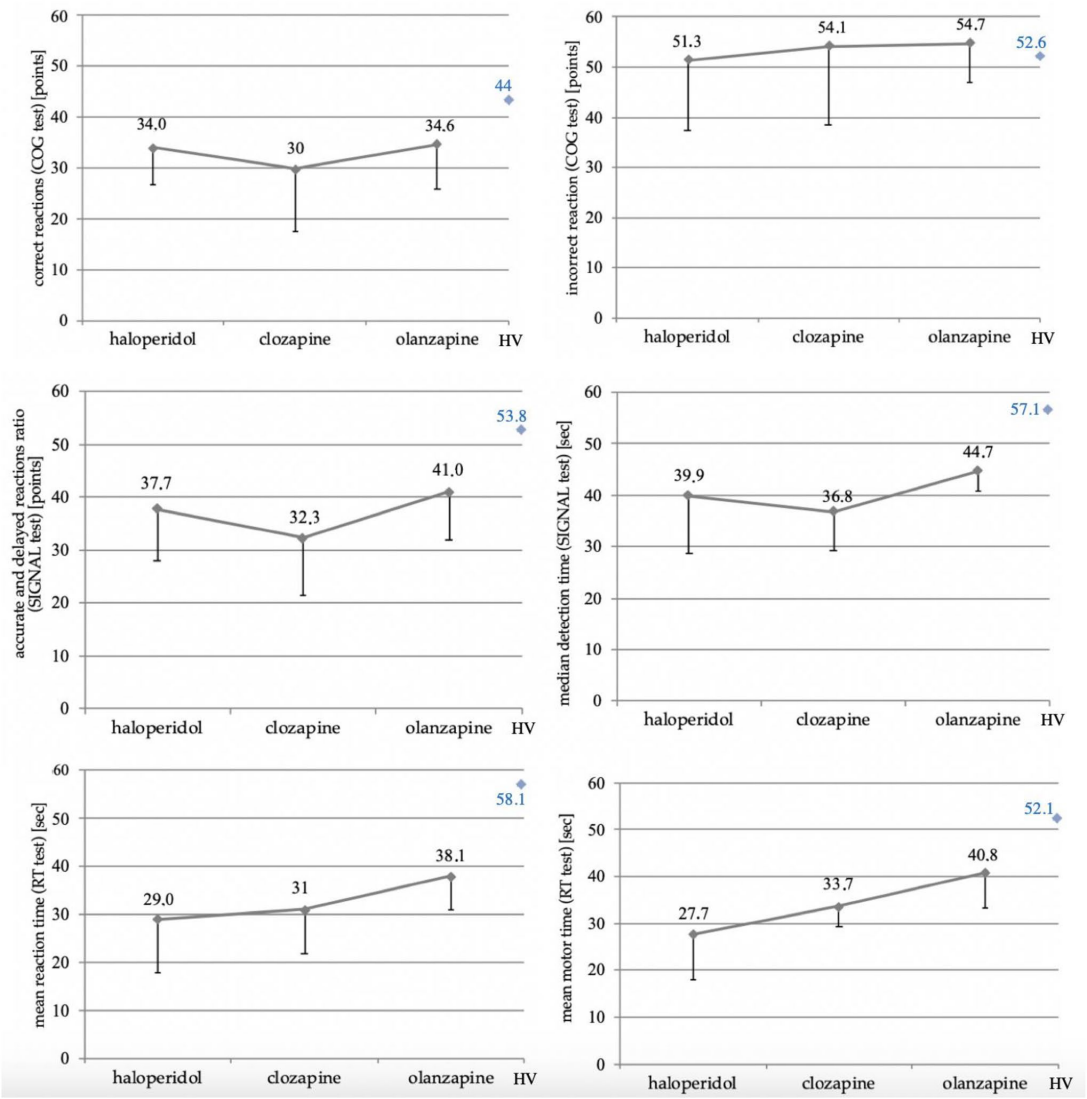
The results of the studied cognitive functions in individual tests were below the normal range, except for incorrect reactions evaluated using the COG test. Description in the text. The results of the studied group of healthy volunteers (HV) were highlighted in blue

Tests belonging to the test system for computerized psychological assessments Vienna Test System (VTS, Schuhfried, Austria) were used to assess cognitive functions of patients [13]. Rated were total correct rejections, total incorrect reactions [with Cognitron (COG) test], the ratio of the number of accurate and delayed reactions, median detection time [with Signal detection (SIGNAL) test], mean reaction time and mean motor time [with Reaction (RT) test]. The results obtained by the patients, in addition to incorrect reactions evaluated using the COG test, were below the normal range. The results on the ten scale (norm for the range of 40–60 points) are presented in Fig. 2.

Analysis of Pearson’s correlation of cognitive test results with demographic and clinical parameters and regression analysis did not show consistent significant correlations, indicating a relationship between them. Below is a brief description of the tests used.

Cognitron (COG) test assesses the concentration performance. Patient compares on the screen a geometric figure with other four geometric figures, stating whether the comparison figure is identical to one of them. Patient has a short time window in which to react only if a figure is identical to the comparison figure. Two values were assessed: total correct rejections, that measures the processing speed and incorrect reactions that measures working accuracy during concentrated working tasks.

Signal detection test (SIGNAL) measures long-term focused attention and the visual differentiation of a relevant signal when distractor signals are present. During the test, the screen shows a display of dots that appear and disappear at random. The patient must react as soon as four dots form a square. With this test, the ratio of the number of accurate and delayed reactions, that is the measure of visual detection performance and median detection time, the measure of the average time taken to make a correct response over the course of the complete test was assessed.

Reaction test (RT) is used to assess the ability to react under simple stimulus constellations (simple and choice reactions). During the test, patient reacts as quickly as they can to optical or acoustic signals. This involves pressing or releasing a button as quickly as possible when a simple light signal (yellow or red light), a tone or a combination of two stimuli (yellow and tone or yellow and red) is presented. Using the RT test, mean reaction time, that indicates the difference in reaction time between tonic and phasic alertness and mean motor time, the indicator of difference in motor time between tonic and phasic alertness was assessed.

### Ethical issues

The study protocol was approved by the Bioethics Committee of the Medical University of Silesia in Katowice No. NN-6501-129/05. Before the enrollment, each patient was given information about the study to read and had the opportunity to ask additional questions. Before enrollment in the study, each patient signed an informed consent to participate in the study.

### DNA isolation

Blood samples for genetic procedures have been collected since 2007 and systematically sequenced up to 2017. The nucleic acids were isolated from the peripheral blood of patients and healthy volunteers using the Blood Mini kit (A&A Biotechnology, Poland) according to the manufacturer’s protocol. Concentrations of isolated DNA samples were measured spectrophotometrically using Biomate 3 spectrophotometer (Thermo Scientific, USA) at wave length 260/280 nm.

### DNA sequence variants detection and analyses

Single nucleotide variants in NMDA-R subunits *GRIN1* (NG_011507.1; Table 2) and *GRIN2B* (NG_031854.2; Table 2) have been previously described in the literature and studied in various populations, as associated with schizophrenia or effectiveness of its treatment [16, 20–23]. The MAF for the first one is only 0.1, but the OD is relatively high: 1.85 for heterozygotes (95% CI 1.43–2.42, *p* < 0.0001) and 2.86 for homozygotes (95% CI 1.17–6.84, *p* = 0.017) [23]. The variants in *GRIN2A* (NG_011812.2; Table 2) and *GRIN*2B (NG_031854.2; Table 2) were determined according to the NMDA-R protein structure available at the time when the research was conducted in The Universal Protein Resource database (https://www.ebi.uniprot.org) and proposed by us as places that may be of key importance for receptor function. Table 2 presents an attempt to reference SNVs from source publications for currently available gene sequences of NMDA-R subunits.

**Table 2:** Single nucleotide variants (SNV’s) selected in the study with their position in reference sequence, location in the gene, putative function and data related to Minor Allele Frequency

Gene/reference sequence number NCBI	dbSNP number/the location of the tested site in the reference sequence	The location of changes in the gene	Function	MAF
*GRIN1*/NG_011507.1 [1001 (C)]	rs11146020/NG_011507.1:g.4476G > C/	5′UTR, the promoter region	Polymorphism associated with susceptibility to schizophrenia	Global—0.107 Europe—0.100
*GRIN2A*/NG_011812.2 [2150–2152 (AAT)]	Lack of described SNV NM_00833.5: c.2304–2306 (AAT)/NG_011812.2:g.358165–358167 NP_000824.1:p.Asn614/	Exon 10, coding sequence, ligand-gated ion channel	Site determining NMDA receptor function	Not applicable
*GRIN2B*/NG_031854.2 [2664 (G)]	rs1806201/NG_031854.2:g.422439C > T NP_000825.2:p.Thr888 =/	Exon 13, coding sequence, synonymous variant	Polymorphism associated with the effectiveness of schizophrenia treatment	Global—0.303 Europe 0.278
*GRIN*2B/NG_031854.2 [2052–2054 (AAC)]	Lack of described SNV NM_000834.5: c.2055-2057 (AAC)/NG_031854.2:g378243–378245 NP_000825.2:p.Asn615/	Exon 9, coding sequence, ligand-gated ion channel	Site determining NMDA receptor function	Not applicable

The original location of SNVs, basing on the source publications are placed in square brackets

*NCBI* National Center for Biotechnology Information, USA, *dbSNP* Single Nucleotide Polymorphism Database of Nucleotide Sequence Variation, *MAF* Minor Allele Frequency

DNA fragments including the studied polymorphisms of the NMDA receptors subunit genes were amplified by polymerase chain reaction (PCR) and analyzed using the ABI Prism 310 capillary sequencer (Applied Biosystem, USA) according to the Applied Biosystem protocol.

### Polymerase chain reaction (PCR)

Forward and reverse primers for PCR were designed based on the nucleotide sequences of the tested genes available in online databases (GeneBank) (Table 3).

**Table 3:** Primer sequences for studied genes of NMDA-R subunits

Gene	Forward primer	Reverse primer
*GRIN1*/NG_011507.1 (subunit NR1)	ACGCGGTGACACGGACCCCTCTAACGT (56 °C)	TCTGTTCGTGTACATGCGTGTGAATGACC (57.8 °C)
*GRIN2A*/ NG_011812.2 (subunit NR2A)	GGGCAATCACAGGACACAACTATC (57.1 °C)	CAGATGGAGAGGAAAGCAAGGTGA (57.1 °C)
*GRIN2B*/NG_031854.2 (subunit NR2B)	AGTTCAAGAAAGACCATCCTACA (54.4 °C)	AAAACATAAGAAAGAACGGTCAAT (54.4 °C)
*GRIN2B*/NG_031854.2 (subunit NR2B)	TGCCCACTTCCAACTCCTACTTAC (56 °C)	CATGATGTGGTTTCTTGCTTGAG (56 °C)

()—The optimal annealing temperature is given in brackets

The PCR reaction was carried out in a 50 µl volume containing 200 nanogram of nucleic acids, Fast Start Taq polymerase (Roche, Germany), 0.25 pmol of each of the primers (Sigma-Aldrich, Poland), a buffer containing MgCl_2_ and 10 mM PCR nucleotide Mix (dNTP’s) (Roche, Germany). The final sample volume was adjusted to 50 µl with deionized water. The amplification reaction was carried out in a Mastercycler thermocycler (Eppendorf, Germany). The samples were denatured initially for 5 min at 94 °C, followed by 29 cycles in the following steps:

- proper denaturation for 30 s at 94 °C,
- annealing for 1 min, at a temperature depending on the starter used (Table 3),
- elongation for 1.5 min at 72 °C.

The final elongation time was 5 min at 72 °C. At the final stage, the samples were cooled to 4 °C.

The 5 μl of the PCR product was separated on 2% ethidium bromide stained agarose gels. The gels were then visualized in ultraviolet light and analyzed using Kodak 1D Image Analysis 3.6 Software.

The sequencing reaction was carried out with Shrimp Alkaline Phosphatase and Exonuclease I (Fermentas, Lithuania) at 37 °C for 15 min and then at 80 °C for 15 min. Sequential PCR was then performed separately for each of the primer pairs using the BigDye Terminator v3.1 Cycle Sequencing Kit (Applied Biosystems, Germany) according to the protocol. The mixture was incubated in a thermocycler for 25 cycles including: denaturation at 96 °C for 30 s, annealing at 50 °C for 15 s, elongation at 60 °C for 3 min. and then cooled at 4 °C. The PCR products were precipitated with alcohol, lyophilized using Speed-Vac (Labconco, USA), and finally dissolved in 20 μl of Hi-Di Formamide solution (Applied Biosystem, USA). The samples sequences were analyzed using ABI Prism 310 capillary analyzer (Applied Biosystem, USA). The results obtained were verified for alignment using MegAlign software, DNAStar 5.0 Software (Lasergene, USA).

### Statistical methods

Descriptive statistics were used in the description of the study groups. Means and standard deviation as well as range of results were used in the calculations. The G*Power program (version 3.1.9.2) was used to perform power analysis [24].

## Results

**Fig. 3:**
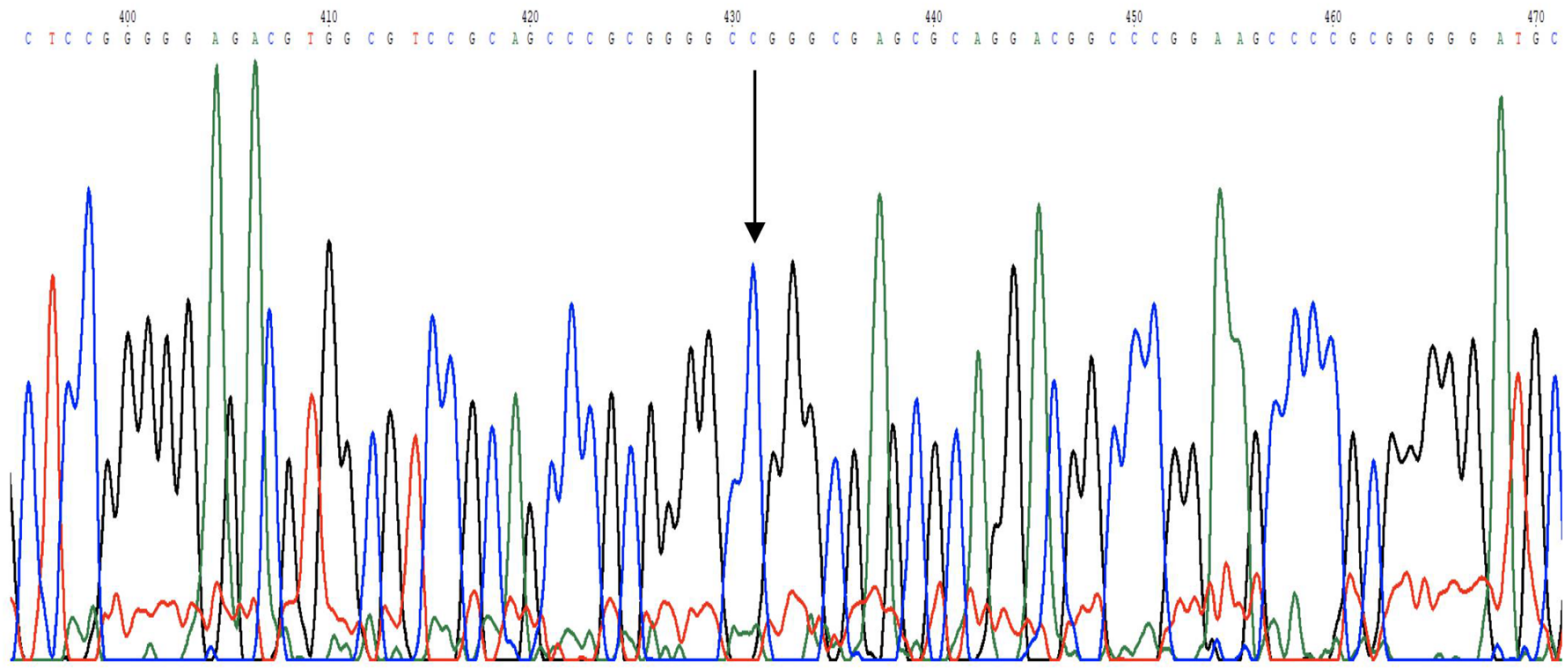
Graphical presentation and alignments of sequences around the selected single nucleotide variants *GRIN1.* The lack of a change in the position 1001 in a comparison to the reference sequence L13266.1 is indicated by arrow

Sequencing of the DNA fragments from schizophrenic patients and healthy control group was conducted to search for a sequence variation in the gene subunit *GRIN1* (rs L13266.1) associated with susceptibility to schizophrenia, *GRIN2B* (rs1806201) associated with the efficacy of neuroleptic and polymorphisms in genes of subunits, which determine NMDA receptor functions—*GRIN2A* [2150–2152 (AAT)] and *GRIN2B*—2052–2054 (AAC). In the studied fragments of human DNA sought SNVs were not detected, both in the group of patients and control group of healthy individuals. An example of a chromatogram showing no change in position 1001 along with a reference sequence is shown in Fig. 3.

**Fig. 4:**
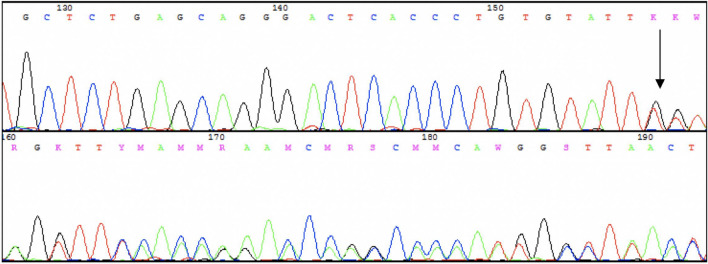
Example of sequencing a fragment of the *GRIN2B* subunit gene with an A deletion in the position 378117 (rs56069446). The deletion site is indicated by an arrow

At the same time, the sequences surrounding target SNVs were analyzed. SNV rs56069446 was found in the intron 8 of the *GRIN2B* gene, present in one healthy person. This A deletion has not been described as clinically relevant to date. The chromatogram marked with the site of this deletion is shown in Fig. 4. The incidence of MAF in the general population is estimated at 0.024 and the European population at 0.075. This change is not yet described, and data on the occurrence of this change are derived from population sequencing (1000 Genomes). Because of too low MAF association studies were not applied.

Power analysis was performed for two SNVs for which MAFs in the European population are known (Table 2). For the SNV in rs1806201 (GRIN2B gene), the MAF value for the European population is 0.278, therefore we assumed the effect size 0.3 in the calculations. To achieve the test power of 0.8 at the significance level *α* = 0.05 and the assumption of a correlation factor of 0.3, the required size of the research group is 64 patients. For our group of 117 patients, under these assumptions, the power of the test is 0.958. In turn, for the SNV in rs11146020 (GRIN1 gene) the MAF value is 0.1, so in the calculations we assumed the effect size 0.1. To achieve the test power of 0.8 with the significance level *α* = 0.05 and the assumption of the correlation factor 0.1, the required size of the research group is 614 patients. For our group of 117 patients, under these assumptions, the power of the test is 0.286.

## Discussion

The hypothesis to verify was whether persistence of cognitive dysfunctions despite the remission of other symptoms of schizophrenia may be a schizophrenia phenotype associated with selected single nucleotide variants in the genes of NMDA-Rs’ subunits. However, we could not confirm this hypothesis. Specifically, the power analysis showed that the group of 117 people would be sufficient to conduct the association study for the SNV in rs1806201 and too small for the SNV in rs11146020. If we were to conduct an associative study in a group of patients with schizophrenia, we would have to gather a sufficiently large study group, especially with regard to SNV prevalence data in rs11146020 in the population. We approached this issue from the other side. We assembled a targeted study group with a selected clinical phenotype, and in this population, we assumed the presence of SNVs in most of the subjects. Our analysis of these SNVs did not show such a relationship.

Thus, observed cognitive impairment in these patients may of course be associated with some other type of genetic conditions, such as the impact of environmental factors or, as we have shown in previous studies, the direct impact of antipsychotic drugs themselves [25, 26]. It is difficult to distinguish the effect of primary NMDA-R dysfunction from the effect that treatment may have on it. This would be possible if a group of people with the first episode of schizophrenia who had not been treated before (drug naive) could be collected and examined.

Although the results of studies are negative, they indicate the need to look for other variants, also within the NMDA receptors subunits and their relationship with schizophrenia. Finding such genetic markers of schizophrenia would be a significant advance in the diagnosis of this disabling and chronic disease as well as in the possibility of early treatment intervention in children and adolescents who would be confirmed by the occurrence of a genetic predisposition to cognitive impairment, which is the clinical manifestation of the primary pathogenetic process in schizophrenia.

In conclusion, selected single nucleotide variants in *GRIN2A* and *GRIN2B* genes of NMDA-R are not associated with the selected clinical phenotype of patients in whom cognitive dysfunctions are present despite effective antipsychotic treatment of other schizophrenia symptoms. The results regarding the SNV in *GRIN 1* are debatable due to the low power of the test and therefore require confirmation in further studies.
